# Odonto-Chirurgical Society of London

**Published:** 1883-05

**Authors:** 


ARTICLE VI.

### ODONTO-CHIRURGICAL SOCIETY OF LONDON.

The Annual Meeting of this Society was held on the 13th ult., at 30, Chambers Street, Edinburgh—Dr. Smith, president, in the chair.

Mr. A. B. Verrier, Weymouth, read a Paper descriptive of his "Gas Furnace and Continuous Gumwork in Connection with Plastic Bases," the various stages of which were demonstrated at 16, George Square, at the close of the meeting.

Mr. Macleod communicated notes on "The Electric Light in Dental Surgery." There was, he said, no doubt that a clear, steady, and diffused light capable of being located within the cavity of the mouth would be a very great advantage to dentist's generally. To those whose misfortune it was to practice their profession in cities which were frequently visited by fogs, it would be a still greater boon. Attention had for some time past been directed to electricity as a commercial source of light, and it might now be looked upon as one of our standard sources, although not yet quite ready to supplant its older rivals, gas and oil. In their specialty, attempts have been made to render it available, and with some success, as the two apparatuses which he brought under the Society's notice would show; but it yet remained for the further development of means to ensure a source of "supply of power" which should be available on a moment's notice, and at the same time comparatively cheap, before they could hope that it would be adopted generally in the Profession, the battery as a source of power being open to the objections of inconstancy, wastefulness, and requiring careful and constant supervision. These faults were certainly considerably modified if, besides the light, the dentist used the mallet or motor, and thus, having the battery in more constant use, utilized the decomposition of material which constantly went on after the battery was charged. What they wanted, however, was an accumulator in which the power might be stored, and drawn from whenever required, and from which none would escape when not in use; or a small dynamo which would generate it cheaply when required, or a general depot from which they could have a wire and turn it on when required, as they did their gas. These were still in the future, and those who wished to avail themselves of the electric light in dentistry must in the meantime be content with the D'Or battery (of which he showed one,) or the bichromate battery, which he also exhibited; and showed by his manipulation of it in the performance of experiments, which were remarkably successful, that it illumined the mouth sufficiently to enable the dentist to almost see into the centre of the teeth.

Mr. Macleod explained the details of a case which he had successfully introduced an artificial obturator and nasal support, which was described by the chairman as one of the most interesting cases of mechanico-surgical Dentisiry on record. Mr. Macleod remarked that the case was a strong example of the beneficial effects upon the Dental Profession by the establishment of a curriculum, and a school to carry out that curriculum.

Mr. Williamson and Mr. Macleod directed attention to "Cases of Deficient Dentition." Mr. Macleod referred to the case of three members of a family who had only twenty-seven teeth amongst them. The eldest girl, 27, had eight permanent teeth; her sister, a young lady of 22, twelve; and her brother, a lad of 17, seven. The two grandmothers of these patients were also deficient in the number of teeth, while the father and mother, and a sister 23 years of age, were normal in their dentition. The father and mother were cousins. In answer to questions, Mr. Macleod said the patients had more than average intelligence. They were what might be called intellectually inclined, two of the sisters having gone through a course of training as nurses, and one of them had married a medical missionary, and gone out to share his labors as well as his lot. They were above the middle-class of society; their physical development was, as far as he could see, in every respect sound; and they were rather good looking.

Mr. Stirling, Ayr, having pointed out improvements which he had made on his articulator.

Mr· Finlasod exhibited a model of the upper and lower teeth of the "Amazon Queen," which, he said, were very small, though she was 8 feet 2 inches in height, and the arch of her mouth very large. The lady had only lost one tooth. Her age was given as 18, but he (Mr. Finlayson) should suppose she was several years more than that.

The Chairman afterwards delivered a valedictory address, in the course of which he congratulated the Society on the work done during his term of office as in every way creditable to any association of the kind, and concluded by introducing his successor as one of the most scientific practitioners in the Dental Profession. Mr. Wilson took the chair, and briefly thanked the Society for the honor conferred upon him. This concluded the business.—*London Dental Record.*

